# Serotonin Associated Mechanisms in Sudden Unexpected Death in Epilepsy: A Review

**DOI:** 10.31083/RN45871

**Published:** 2026-03-24

**Authors:** Beixu Li, Wenrui Zhao, Junyi Lin, Yue Chen, Ru Zhou, Kaijun Ma, Youxin Fang

**Affiliations:** ^1^School of Forensic Science, Shanghai University of Political Science and Law, 201701 Shanghai, China; ^2^Shanghai Fenglin Forensic Center, 200231 Shanghai, China; ^3^Shanghai Key Laboratory of Crime Scene Evidence, Institute of Forensic Science, Shanghai Public Security Bureau, 200083 Shanghai, China; ^4^School of Forensic Medicine and Science, Fudan University, 200032 Shanghai, China; ^5^Department of General Surgery, Ruijin Hospital Luwan Branch, School of Medicine, Shanghai Jiaotong University, 200020 Shanghai, China; ^6^Department of Neurology, Minhang Hospital, Fudan University, 201100 Shanghai, China

**Keywords:** serotonin, 5-HT, epilepsy, sudden unexpected death in epilepsy, autonomic nervous system

## Abstract

Sudden Unexpected Death in Epilepsy (SUDEP) is the leading cause of death in patients with epilepsy (PWE), although the mechanisms are unclear. Early studies have shown that abnormal cardiopulmonary function plays a key role in SUDEP. Cardiopulmonary activity is regulated by the autonomic nervous system. Serotonin (5-hydroxytryptamine or 5-HT) neurons significantly influence respiration and are also closely related to epilepsy. Therefore, serotonin is hypothesized to be involved in SUDEP, and a substantial amount of research has focused on it. Notably, serotonin signals through at least 14 known receptor subtypes, with preclinical data suggesting a particular involvement of the 5-HT_2_, 5-HT_3_, and 5-HT_4_ receptors in SUDEP. Dilute Brown Non-Agouti (DBA)/1 and DBA/2 mice, which often die of seizure-induced respiratory arrest (S-IRA) following audiogenic seizures (AGS), are the most commonly used animal models for studying SUDEP. Increased serotonin reduces S-IRA, activating serotonin neurons prevents SUDEP, abnormalities in serotonin receptors are associated with SUDEP, and selective serotonin reuptake inhibitors (SSRIs) affect electroencephalogram (EEG) activity. Other studies have found that serotonin protects against S-IRA in PWE. Pathological studies in patients with SUDEP have also revealed that, in comparison with controls, the axonal length (AL) of serotonin transporter (SERT)-positive axons is longer and the level of tryptophan hydroxylase (TPH), the rate-limiting enzyme in serotonin synthesis, is lower. Consequently, serotonin is possibly a potential target for preventing SUDEP. However, most of the results are from animal studies, while the experimental data in PWE are limited. More human studies are needed in the future.

## 1. Introduction

Epilepsy is a chronic brain disorder characterized by recurrent hypersynchronous neuronal discharges. As a prevalent neurological disorder, it is one of the most common nervous system diseases. Its prevalence is approximately 4.9‰ in developed countries and 12.7‰ in developing countries [1]. An epidemiological survey conducted by the World Health Organization and China in 2001 showed that the lifetime prevalence of epilepsy was 7‰ in China, and an estimated 9 million people, were suffering from epilepsy in China at that time. Sudden unexpected death in epilepsy (SUDEP) is defined as a death in a patient with epilepsy that is not directly due to status epilepticus, trauma, or drowning, occurs in the context of a seizure, and for which no clear cause of death is revealed by autopsy and toxicological analysis [2]. SUDEP accounts for 17% of all deaths in patients with epilepsy (PWE) and is the leading cause of death in this population. Its incidence in PWE exceeds the rate of sudden death in the general population by 20 times [3, 4, 5]. The sudden and unexpected nature of SUDEP imposes a profound and lasting psychological burden on families and caregivers, compounding the existing challenges of managing a chronic neurological condition. This significant human impact underscores the critical need for research aimed at elucidating its mechanisms and developing effective preventive strategies.

Though the exact mechanism of SUDEP still remains unclear, three widely-acknowledged risk factor groups have been identified. First, seizure-related factors: the occurrence of generalized tonic-clonic seizures (GTCS) is the most significant and consistent risk factor. Patients with three or more GTCS per year are at a substantially higher risk. Second, demographic and comorbidity factors: although SUDEP can occur at any age, incidence peaks in young adulthood (20–40 years), which may be related to lifestyle factors. The presence of developmental or intellectual disabilities is also a significant risk factor. Third, genetic and physiological factors, including genetic mutations, prone sleeping position, central apnea, and cardiac arrhythmia, etc. [6, 7]. Early studies found that abnormal cardiopulmonary function plays an important role in SUDEP. Simultaneous electroencephalography (EEG)-electrocardiography (ECG) monitoring in PWE has shown that various arrhythmias, such as tachycardia, bradycardia, and sinus arrest, may occur during seizures, accompanied by central apnea [8, 9]. Forensic studies of SUDEP have found similar results. A study in 1908 reported that pulmonary edema was commonly found in PWE at autopsy [10]. In 2012, American forensic scientists reported that SUDEP individuals showed left ventricular hypertrophy (9.5%) and atherosclerotic coronary artery disease (4.1%) at autopsy, and myocyte hypertrophy (21.2%) and focal fibrosis in the myocardium (42.3%) upon microscopic examination; all these subjects showed pulmonary congestion and edema to varying degrees [11]. Abnormal cardiopulmonary function is likely to be a direct cause of SUDEP, though this is hard to confirm since there is typically no immediately lethal pathological change identified.

A substantial body of clinical and basic research has consequently focused on the role of serotonin in SUDEP. In this review, we will summarize the advances in understanding serotonin-associated mechanisms in SUDEP.

## 2. Methods

This narrative review was conducted to appraise the existing literature on the role of serotonin in SUDEP. A systematic search was performed to ensure a comprehensive and reproducible collection of relevant studies by BX L, WR Z, JY L.

The methodology involved a systematic search of academic databases, including PubMed/MEDLINE (https://pubmed.ncbi.nlm.nih.gov/https://www.nlm.nih.gov/medline/index.html) and Web of Science (Core Collection, https://www.webofscience.com), to identify pertinent publications. The search was structured using a combination of the following key terms: (“sudden unexpected death in epilepsy” OR “SUDEP”) AND (“serotonin” OR “5-HT” OR “5-hydroxytryptamine”). Additional focused searches were performed using the terms: (“seizure-induced respiratory arrest” AND “serotonin”) and (“epilepsy” AND “serotonin” AND “respiration”). The publication date filter was set from database inception until December 2025 (**Supplementary Fig. 1**).

Articles were first screened based on their titles and abstracts to assess their relevance to the core themes of the review. Potentially relevant articles were then obtained in full text and evaluated against predefined inclusion and exclusion criteria (**Supplementary Table 1**). Studies not focused on serotonin’s role in epilepsy or SUDEP, or with no relevant outcomes, were excluded. To mitigate the risk of missing important references, the reference lists of key review articles and eligible primary studies were manually screened for additional relevant publications.

The methodology, while systematic, has inherent limitations. As a narrative review, it does not claim to be exhaustive of all existing literature. The focus on major databases and English-language publications may have introduced selection bias. Furthermore, the broad and interdisciplinary nature of the topic means that some relevant studies in specialized fields might have been overlooked despite our comprehensive search strategy.

## 3. Serotonin and Respiration Function

Cardiopulmonary activity is regulated by the autonomic nervous system. The central autonomic network (CAN) is a circuit extending from forebrain to brainstem, in which central information is transmitted from the insula, medial frontal cortex, and hippocampus to the hypothalamus, pons and medulla oblongata, then it regulates autonomic nervous activity through efferent nerves [12]. Since some components of the CAN may be the origin of epileptic discharge (e.g., hippocampus) or a link of epileptogenic network (e.g., hypothalamus), seizures can be accompanied by autonomic symptoms, such as salivation, piloerection, and urination, and can also lead to abnormal cardiopulmonary function [13].

Serotonin (5-hydroxytryptamine or 5-HT) is a monoamine neurotransmitter. Central serotonin neurons are primarily located in the raphe nuclei of the brainstem, whose projections reach respiratory-related nuclei within the CAN, such as the nucleus of the solitary tract (NTS), the retrotrapezoid nucleus (RTN), and the nucleus ambiguous (NA) [14]. Serotonin neurons have a definite effect on respiration. They not only act as chemoreceptors to stimulate breathing when carbon dioxide levels are elevated in the body [15], but also mediate respiratory plasticity during intermittent hypoxia [16]. Lesions of the respiratory-related nuclei that receive serotonergic projections result in respiratory depression, while serotonin receptor agonists stimulate breathing and reduce the respiratory inhibition effect caused by certain sedatives [17, 18]. Due to the close connection between serotonin neurons and respiration, researchers have suggested that serotonin may be related to sudden infant death syndrome (SIDS) [19, 20, 21]. Studies in SIDS individuals have found elevated serum level and an increased number of serotonin neurons in brainstem, while the number of 5-HT receptors is insufficient and the receptor binding rate is decreased.

Research has identified that specific serotonin receptor subtypes are critically involved in these protective respiratory mechanisms. Notably, the 5-HT_2A_ receptor has been identified as an important mediator of CO_2_-induced arousal from sleep, a key protective mechanism pertinent to SUDEP in animal models [22]. Evidence from both human studies and animal models indicates that multiple serotonin receptor subtypes work in concert to maintain respiratory stability during the vulnerable period following a seizure. Enhancing serotonergic tone, potentially by targeting specific receptors, presents a promising therapeutic strategy for mitigating seizure-induced respiratory arrest (S-IRA) and preventing SUDEP [23].

## 4. Serotonin and Epilepsy

Serotonin is also closely associated with epilepsy. Researches on different types of epilepsy animal models have found that selective serotonin reuptake inhibitors (SSRIs) can reduce seizure frequency by boosting its concentration in synapses [24, 25].

Furthermore, numerous studies have investigated the relationship between 5-HT receptors and epilepsy. 5-HT receptors consist of seven types, 5-HT_1_ through 5-HT_7_, and fourteen subtypes. All of these types except 5-HT_5_ have been implicated in epilepsy. However, these findings are often model-specific and contradictory data exist, underscoring the importance of distinguishing between preclinical mechanisms and translatable human therapeutic targets (Table 1, Ref. [26, 27, 28, 29, 30, 31, 32, 33, 34]). We have evaluated the strength and translatability of evidence by prioritizing human data, assessing reproducibility across models, weighing the consistency of findings, and considering biological mechanisms.

**Table 1.** **Key preclinical and clinical evidence for the most studied 5-HT receptor subtypes in epilepsy**.

5-HT receptor subtype	Role in seizures	Key supporting findings and level of evidence	References
5-HT_1A_	Anticonvulsant (in most models except absence epilepsy)	Agonists (e.g., 8-OH-DPAT) suppress seizures in focal models. Human PET shows decreased binding in TLE, independent of depression. Density in the hippocampus positively correlates with epilepsy duration, possibly due to compensatory upregulation.	[26, 30, 31]
		Moderate evidence; reproducible human imaging finding	
5-HT_1D_	Anticonvulsant	Agonists reduce seizures in zebrafish Dravet syndrome models. A drug repurposing screen in the same model identified compounds with antiseizure activity, including one that demonstrated efficacy in a rodent model of drug resistant epilepsy	[27]
		Promising but preliminary evidence; reproducible in specific gene models	
5-HT_2A_	Dual/Context-dependent role	Can be proconvulsant (facilitates kindling at high doses). However, it is critically anticonvulsant and anti-SUDEP: agonists prevent S-IRA and mediate CO_2_-induced arousal. It is a direct target of the antiseizure medication (ASM) fenfluramine.	[29, 32]
		Conflicting evidence; low reproducibility across different models	
5-HT_3_	Anticonvulsant (predominantly)	Agonists (e.g., SR 57227) decrease seizure scores and mortality in acute models. Likely acts on inhibitory interneurons to reduce network excitation.	[28]
		Consistent pharmacological evidence in acute models	
5-HT_4_	Modulatory (evidence is contradictory)	Some data suggest antagonists are anticonvulsant, while other studies show agonists (potentially via fenfluramine) may protect against SUDEP.	[32]
		Conflicting evidence; low reproducibility across different models	
5-HT_6_	Proconvulsant	Antagonists reduce seizures in chronic models. Upregulated in the hippocampus of patients with drug-resistant TLE.	[33]
		Emerging but inconclusive evidence; clear translational disconnect between animal and human tissue studies	
5-HT_7_	Proconvulsant (predominantly)	Antagonists are anticonvulsant in multiple models. Upregulated in the neocortex of patients with drug-resistant TLE.	[34]
		Emerging but inconclusive evidence; clear translational disconnect between animal and human tissue studies	

8-OH-DPAT, PET, positron emission computed tomography; TLE, temporal lobe epilepsy; S-IRA, seizure-induced respiratory arrest.

For instance, 8-OH-DPAT, a 5-HT_1A/7_ receptor agonist, was shown to improve spike-wave discharges in rat models of focal epilepsy [26]. 5-HT_1D_ receptor agonists reduced seizure frequency in zebrafish models of drug resistant epilepsy [27]. SR 57227, a 5-HT_3_ receptor agonist, decreased seizure score and mortality in mouse models of pentylenetetrazole (PTZ)-induced seizures [28]. Drugs acting on the 5-HT_2A_ receptor affected seizure onset in animal models of temporal lobe epilepsy (TLE) [29].

In addition to animal research, some studies on PWE have also revealed associations between epilepsy and serotonin. A positron emission computed tomography (PET) study found a significant decrease of 5-HT_1A_ receptor binding proteins in the temporal lobe of patients with temporal lobe epilepsy, with no association between this reduction and depression scores [30]. A study on surgical specimens from patients with drug-resistant TLE found that the low serotonin level in temporal lobe was related to a history of GTCS, but not to mental illness [35]. The density of 5-HT_1A_ receptors in the hippocampus was positively associated with epilepsy duration [31], possibly due to the upregulation of the receptor resulting from the prolonged and enduring epileptic activity.

## 5. Serotonin and SUDEP

**Fig. 1.**
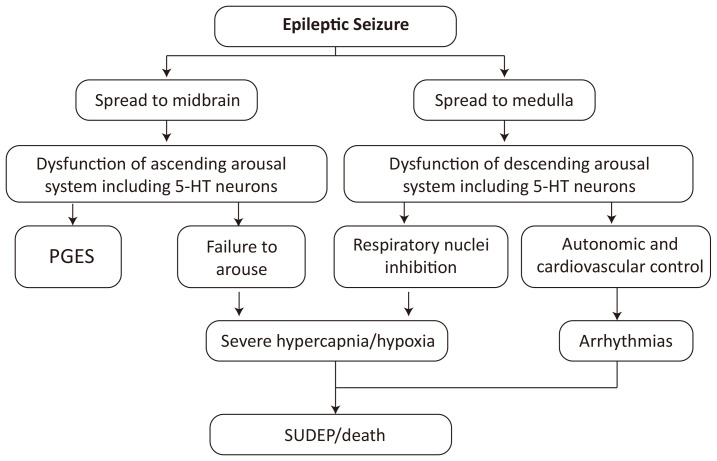
**Hypothesis of SUDEP mechanism [36]**. SUDEP, Sudden Unexpected Death in Epilepsy; PGES, postictal generalized EEG suppression. EEG, electroencephalography; 5-HT, 5-Hydroxytryptamine.

Massey *et al*. [36] proposed a hypothesis of SUDEP mechanism: aberrant discharges spread to the ascending arousal system containing serotonin neurons in the midbrain, leading to failure of arousal in prone patients and eventually resulting in asphyxiation; the spread of aberrant discharges to the descending neural pathway containing serotonin neurons in the medulla oblongata inhibits respiratory-related nuclei and affects the regulation of cardiac autonomic nerve, thus leading to hypoventilation, arrhythmias, and even death (Fig. 1, Ref. [36]).

There have been many animal studies on the relationship between serotonin and SUDEP. DBA/1 and DBA/2 mice are the most commonly used animal models for SUDEP, in which audiogenic seizures (AGS)—GTCS induced by 95–122 dB sound stimulation—occur, and the mice often die of S-IRA after such seizures [37, 38].

First, increased serotonin has been found to reduce the incidence of S-IRA in animal models. Tryptophan generates 5-hydroxytryptophan in the presence of tryptophan hydroxylase (TPH), which is then converted to serotonin by 5-hydroxytryptophan decarboxylase. The metabolic end product of serotonin is 5-hydroxyindoleacetic acid (5-HIAA). TPH-2 is the rate-limiting enzyme for serotonin synthesis in the central nervous system [39]. One study reported decreased levels of TPH-2 protein expression in the brainstems of DBA/1 mice [40]. Another study found declined TPH-2 activity and reduction of 5-hydroxytryptophan, 5-HT, and 5-HIAA in the brainstems of DBA/1 mice [41]. Research reveals that fenfluramine’s antiseizure and anti-SUDEP effects stem from a complex, dose-dependent pharmacology that involves multiple serotonin receptor subtypes, its metabolite norfenfluramine, and non-serotonergic pathways [42]. Fenfluramine primarily increases synaptic serotonin by promoting its release and inhibiting reuptake via the serotonin transporter (SERT) [43]. In the DBA/1 mouse model, this leads to dose-dependent protective effects [44]. A dose of 15 mg/kg selectively blocks S-IRA without reducing convulsive behaviors. Higher doses (20–40 mg/kg) produce broader anticonvulsant effects, significantly reducing both seizure incidence/severity and S-IRA susceptibility. These effects are mediated by the activation of specific 5-HT receptors by the elevated serotonin. Crucially, the selective blockade of S-IRA is mediated primarily by 5-HT_4_ receptor activation. Studies show that a 5-HT_4_ receptor antagonist can reverse fenfluramine’s protective effect, while a 5-HT_4_ agonist can mimic it [45]. The antiseizure activity, however, involves a broader receptor profile. The active metabolite norfenfluramine is a potent agonist at 5-HT_2C_ and 5-HT_2B_ receptors [46]. 5-HT_2C_ agonism is strongly linked to anticonvulsant effects, while historically, 5-HT_2B_ agonism was associated with valvulopathy—a risk mitigated in current low-dose epilepsy therapy. The translational application of serotonergic drugs like fenfluramine necessitates vigilant safety monitoring. Key concerns include the potential for drug-drug interactions (e.g., with other serotonergic agents or ASMs) [47], the risk of serotonin syndrome, and the requirement for cardiovascular screening due to the historic link between 5-HT_2B_ receptor activation and valvulopathy [48]. These factors underscore the complexity of repurposing potent neuromodulatory drugs for chronic use in a vulnerable patient population.

Furthermore, some animal studies have investigated the relationship between serotonin neurons and SUDEP. Optogenetic activation of serotonin neurons in the dorsal raphe (DR) decreased the incidence of S-IRA, which was reversed by ondansetron, a 5-HT_3_ receptor antagonist [49]. In consequence, activating serotonin neurons protected against SUDEP.

Studies have also conducted on the association between abnormalities in different 5-HT receptor subtypes and SUDEP. Uteshev *et al*. [50] found abnormal expression of 5-HT receptors in brainstems of DBA/2 mice, in which 5-HT_2C_, 5-HT_3_, and 5-HT_4_ receptors (with excitatory effects towards respiratory) were decreased, while 5-HT_2B_ receptor (with inhibitory effects) were enhanced. 5-HT receptor antagonists and agonists affect the incidence of S-IRA. Ma *et al*. [51] reported that ketanserin, a 5-HT_2_ receptor agonist, increased the incidence of S-IRA in DBA/1 mice. Another study proved that inflammation induced by lipopolysaccharide had a protective effect against S-IRA in DBA/1 mice, which was blocked by selective 5-HT receptor antagonist cyproheptadine and 5-HT_3_ receptor antagonist ondansetron [52]. Faingold *et al*. found that SR 57227, a 5-HT_3_ receptor agonist, had a similar effect with fluoxetine in reducing the incidence of S-IRA [53]. Therefore, SUDEP is possibly associated with the declined levels of 5-HT receptors with excitatory effects towards respiration and enhanced levels of 5-HT receptors with inhibitory effects in brainstems [54].

There are also several other animal models of SUDEP, such as *Lmx1b*^f/f/p^ mice. In these mice, knockout of the transcription factor Lmx1b results in the specific loss of central serotonin neurons [55]. Buchanan *et al*. [56] demonstrated that *Lmx1b*^f/f/p^ mice subjected to maximal electro-shock (MES) or pilocarpine-induced seizures exhibited a relatively lower seizure threshold and a significantly higher incidence of seizure-related mortality from respiratory failure compared to *Lmx1b*^f/f^ controls. Importantly, this fatal respiratory arrest was shown to be preventable by the stimulation of 5-HT_2A_ receptors, highlighting the receptor’s specific role in protecting cardiorespiratory function post-seizure. Supporting this, the study found that citalopram, a selective serotonin reuptake inhibitor, reduced mortality in *Lmx1b*^f/f^ mice instead of *Lmx1b*^f/f/p^ mice, directly linking the survival benefit to intact serotonergic neurotransmission [56]. While citalopram depends on the presence of functionally intact serotonin neurons to increase extracellular serotonin, the *Lmx1b*^f/f/p^ mouse model demonstrates that this prerequisite is compromised when serotonin neurons are genetically ablated. In contrast, (4-Bromo-3,6-dimethoxybenzocyclobuten-1-yl)methylamine hydrobromide (TCB-2), a selective 5-HT_2A_ receptor agonist, directly stimulates the 5-HT_2A_ receptor, which the research identifies as a critical mediator for protecting cardiorespiratory function post-seizure [57]. This direct agonist action provides a more robust and guaranteed activation of the specific receptor pathway necessary to prevent fatal respiratory arrest, independent of the integrity of presynaptic serotonin synthesis or release. Therefore, TCB-2’s mechanism represents a targeted pharmacological intervention that is effective even in a context of profound central serotonin deficiency, where SSRIs would be expected to fail.

Studies on EEG have also yielded some discoveries. Postictal generalized EEG suppression (PGES) is a period of low-amplitude, low-frequency electrographic activity following some seizures, regarded as a risk marker of SUDEP [58, 59]. An animal study revealed that SSRIs reduced the occurrence of PGES [60]. Leitner *et al*. [61] studied preoperative EEG and surgical specimens from patients with temporal lobe epilepsy, and found that the expression level of 5-HT_2A_ in the hippocampus was significantly positively correlated with the duration of PGES.

Research in PWE also supports the association between serotonin and SUDEP. SUDEP has been linked to ictal central apnea (ICA) [62] and postconvulsive central apnea (PCCA) [63]. ICA is usually recovered quickly, self-limited and benign [64], whereas PCCA has a lower incidence but is more likely to result in SUDEP [65]. In PWE with ICA and PCCA, no difference was found between the blood concentration of serotonin during and after seizures, while the concentration after seizures was significantly higher than during seizures in PWE without central apnea, indicating a protective role of serotonin against respiratory arrest [66]. Another study found that the risk for ICA in PWE treated with long-term SSRIs was significantly lower than in a control group, while there was no difference in the risk for PCCA [67].

Relevant findings have also emerged from pathological studies of surgical and autopsy specimens from PWE. Patodia *et al*. [68] detected the immunohistochemistry labelling index (LI) and axonal length (AL) of SERT-positive axons in surgical specimens from patients with drug-resistant temporal lobe epilepsy and cadaveric specimens from SUDEP individuals. The results showed that SERT LI was significantly higher in high risk SUDEP group than in control group, with the possible mechanism being that SERT is regulated by seizures and serotonergic axons have the potential for repair and remodeling after seizures [68]. Another study from this research team on cadaveric specimens from SUDEP individuals found that, compared to a non-epilepsy control group, the level of TPH2 positive cells in SUDEP group and epilepsy control group was markedly decreased, especially in the ventrolateral medulla region, and TPH2 neurons expressing SERT were reduced in SUDEP individuals, suggesting the probable serotonin re-uptake failure [69].

## 6. Conclusion

Serotonin is a neurotransmitter with clear effects on respiratory function and sleep-wake regulation and is also associated with epilepsy. It is commonly recognized that inhibition of cardiopulmonary function plays a part in SUDEP. Consequently, there have been a large number of researches on the role of serotonin in SUDEP in recent years, yielding some positive results. Certain drugs have been found to affect the incidence and severity of SUDEP by regulating serotonin concentration or targeting 5-HT receptors. However, most of the results are from animal studies, and a critical synthesis of this preclinical evidence reveals significant methodological challenges. These include the use of diverse animal models (acute versus chronic, genetic versus induced), ligands with varying selectivity, and dose-dependent effects, all of which impact the reproducibility and generalizability of findings. Consequently, the most robust conclusions are currently drawn from results consistent across multiple models or supported by direct human evidence, whereas experimental data from PWE remain limited. Priority should be given to neuropathological examinations of serotonergic nuclei within the brainstems of postmortem SUDEP cases. Such studies could provide direct biological validation of receptor abnormalities observed in animal models, with a focus on the density and integrity of serotonin neurons, receptor expression, and transporter levels in key respiratory nuclei. Furthermore, prospective clinical cohort studies in high-risk PWE are essential. These should integrate long-term monitoring of cardiorespiratory function during seizures with biomarkers of serotonergic function to establish correlative risk profiles. Finally, the most definitive, though complex, evidence would come from well-designed interventional trials investigating whether adjunctive serotonergic medications (such as SSRIs) can mitigate peri-ictal respiratory depression or reduce SUDEP incidence in select populations.

In parallel to pharmacological strategies, technological approaches such as wearable seizure detection devices offer a pragmatic, complementary avenue for SUDEP risk mitigation. These devices, which monitor parameters like electrodermal activity, motion, and heart rate, aim to alert caregivers to prolonged or severe convulsive seizures, which are the primary risk factor for SUDEP. While they do not prevent the underlying pathophysiology, they enable timely intervention, potentially interrupting the cascade of events that can lead to fatal cardiorespiratory arrest.

Finally, any future pharmacological strategy targeting the serotonergic system for SUDEP prevention must be developed with a paramount focus on safety. A comprehensive evaluation of long-term tolerability, interaction profiles with common antiseizure medications, and specific risks such as serotonin syndrome or cardiovascular sequelae in people with epilepsy is essential before clinical adoption can be widely recommended [70].

## Supplementary Material

Supplementary material associated with this article can be found, in the online version, at https://doi.org/10.31083/RN45871.
