# Supplementary material for: Serotonin Associated Mechanisms in Sudden Unexpected Death in Epilepsy: A Review
Source: Rev Neurol. 2026 Mar 24;81(3):45871. doi: 10.31083/RN45871 (PMC13036788; doi:10.31083/RN45871)
Supplement: Supplementary file 1 [file 1576-6578-81-3-45871-s1.zip › Supplementary Material.docx]

**Identification of studies via databases and registers**

Records removed *before screening*:

Duplicate records removed manually (n = 628)

Records marked as ineligible by automation tools (n = 0)

Records identified from:

Pubmed/MEDLINE (n = 905)

Web of Science (n = 1460)

**Identification**

Records screened

(n = 1737)

Records excluded for not meeting all inclusion criteria

(n = 1563)

Reports sought for retrieval

(n = 174)

Reports not retrieved

(n = 29)

**Screening**

Reports assessed for eligibility

(n = 145)

Reports excluded:

Wrong article type (n = 18)

Not in English (n = 57)

Studies included in review

(n = 70)

**Included**

Source: Page MJ, et al. BMJ 2021;372:n71. doi: 10.1136/bmj.n71.

This work is licensed under CC BY 4.0. To view a copy of this license, visit <https://creativecommons.org/licenses/by/4.0/>

Supplementary Fig. 1. Flow diagram of the systematic review process. The figure was drawn in Adobe Illustrator 26.4.1.

Supplementary Table 1. Inclusion and exclusion criteria.

| Inclusion | Exclusion |
| --- | --- |
| Peer-reviewed literature | Reporting other specialities than serotonin’s role in epilepsy or SUDEP, or with no relevant outcomes |
| Literature published until December 2025 | Non-research paper: discussion paper, editorial |
| Original research (preclinical & clinical), reviews, and meta-analyses directly investigating serotonin’s role in epilepsy or SUDEP | Abstracts only or inaccessible full text |
| Human studies (PWE, post-mortem studies) and animal models of epilepsy or SUDEP | Non-English publications |
